# Basal progenitor cells in the embryonic mouse thalamus - their molecular characterization and the role of neurogenins and Pax6

**DOI:** 10.1186/1749-8104-6-35

**Published:** 2011-11-11

**Authors:** Lynn Wang, Krista K Bluske, Lauren K Dickel, Yasushi Nakagawa

**Affiliations:** 1 Department of Neuroscience, Developmental Biology Center and Stem Cell Institute, University of Minnesota, Minneapolis, MN 55455, USA; 2 Graduate Program in Neuroscience, University of Minnesota, Minneapolis, MN 55455, USA; 3 Department of Neuroscience, University of Texas Southwestern Medical Center, Dallas, TX 75390, USA

## Abstract

**Background:**

The size and cell number of each brain region are influenced by the organization and behavior of neural progenitor cells during embryonic development. Recent studies on developing neocortex have revealed the presence of neural progenitor cells that divide away from the ventricular surface and undergo symmetric divisions to generate either two neurons or two progenitor cells. These 'basal' progenitor cells form the subventricular zone and are responsible for generating the majority of neocortical neurons. However, not much has been studied on similar types of progenitor cells in other brain regions.

**Results:**

We have identified and characterized basal progenitor cells in the embryonic mouse thalamus. The progenitor domain that generates all of the cortex-projecting thalamic nuclei contained a remarkably high proportion of basally dividing cells. Fewer basal progenitor cells were found in other progenitor domains that generate non-cortex projecting nuclei. By using intracellular domain of Notch1 (NICD) as a marker for radial glial cells, we found that basally dividing cells extended outside the lateral limit of radial glial cells, indicating that, similar to the neocortex and ventral telencephalon, the thalamus has a distinct subventricular zone. Neocortical and thalamic basal progenitor cells shared expression of some molecular markers, including *Insm1*, Neurog1, Neurog2 and NeuroD1. Additionally, basal progenitor cells in each region also expressed exclusive markers, such as Tbr2 in the neocortex and Olig2 and Olig3 in the thalamus. In *Neurog1*/*Neurog2 *double mutant mice, the number of basally dividing progenitor cells in the thalamus was significantly reduced, which demonstrates the roles of neurogenins in the generation and/or maintenance of basal progenitor cells. In *Pax6 *mutant mice, the part of the thalamus that showed reduced Neurog1/2 expression also had reduced basal mitosis.

**Conclusions:**

Our current study establishes the existence of a unique and significant population of basal progenitor cells in the thalamus and their dependence on neurogenins and Pax6. These progenitor cells may have important roles in enhancing the generation of neurons within the thalamus and may also be critical for generating neuronal diversity in this complex brain region.

## Background

The immense number of neurons in the mammalian neocortex is thought to be determined during development by a prominent progenitor cell population that shows a distinct pattern of migration and division. Unlike the predominant progenitor cell type in other brain regions, the radial glial cells (RGs), these cells divide basally away from the ventricular surface and undergo a symmetric division that generates two neurons or two progenitor cells. It is thought that the six-layered mammalian neocortex is largely dependent on division of these basal progenitor cells that serve as transit amplifying cells or intermediate progenitor cells (IPCs), and that the evolution of the mammalian cortex is correlated with the emergence of progenitor cell populations that enhance the generation of neurons [1-4].

Basally dividing progenitor cells have been identified not only in the cerebral cortex, but also in ganglionic eminences, thalamus, hindbrain and spinal cord [5-9]. However, only the cerebral cortex and ganglionic eminences have been shown to harbor a robust enough population of basal progenitor cells to form a distinct domain, the subventricular zone (SVZ), above the domain of RGs that comprises the ventricular zone (VZ). Recent studies identified a number of molecular markers of basal progenitor cells in the developing neocortex. In addition, genes such as *Tbr2 *[10,11], *Insm1 *[12] and *AP2γ *[13] or inhibition of Notch signaling [14,15] are found to be essential for the generation of basal progenitor cells from RGs. Time-lapse analysis of fluorescently labeled cortical progenitors in slices elucidated the unique migratory patterns and modes of division of neocortical basal progenitor cells and showed that these cells function as transit amplifying progenitor cells, or IPCs [9,16,17].

The mammalian thalamus has an extremely complex organization with several dozen distinct neuronal populations called nuclei [18]. During embryogenesis, the thalamus is composed of two molecularly distinct domains of neural progenitor cells, pTH-C and pTH-R, located across rostro-caudal and dorso-ventral axes [19]. pTH-C is a larger, caudo-dorsally located domain that expresses the basic helix-loop-helix (bHLH) transcription factors neurogenin 1 (Neurog1) and neurogenin 2 (Neurog2) and gives rise to all of the thalamic nuclei that project to the cortex. pTH-R is a smaller domain that expresses another bHLH protein, Ascl1 (also known as Mash1), and lies between pTH-C and the zona limitans intrathalamica (ZLI), the boundary population that abuts the thalamus and the prethalamus [19]. pTH-R likely contributes to the majority of GABAergic neurons in the thalamus, including part of the ventral lateral geniculate nucleus and intergeniculate leaflet. Recent studies have unveiled critical roles of secreted signaling molecules in the formation of positional diversity of thalamic progenitor cells [20-24].

Despite the finding that there are basally dividing cells in embryonic mouse thalamus [6], their molecular characteristics and the mechanisms for their generation have not yet been determined. Considering the extensive connections between the thalamus and neocortex, we anticipated that the mammalian thalamus has diversified its progenitor cell populations during evolution to allow generation of a larger number of neurons comparable to those found in the six-layered neocortex.

In the study described here, we explored this possibility by performing a detailed characterization of thalamic basal progenitor cells in mouse embryos. We found that the thalamus contains a remarkably large number of basal progenitor cells, some of which form the SVZ similar to that found in the neocortex and ventral telencephalon. Thalamic basal progenitor cells do not express the same molecular markers as neocortical IPCs, such as Tbr2, but they do share many other aspects with their putative cortical counterpart, including the expression of the bHLH transcription factors NeuroD1 and neurogenins (Neurog1 and Neurog2). We also show the first evidence that Neurog1 and Neurog2 are required for the normal number of basally dividing cells, which demonstrates the critical role of these transcription factors in the formation and/or the maintenance of thalamic basal progenitor cells.

## Results

### The embryonic mouse thalamus contains a large number of basally dividing cells

**Figure 1:**
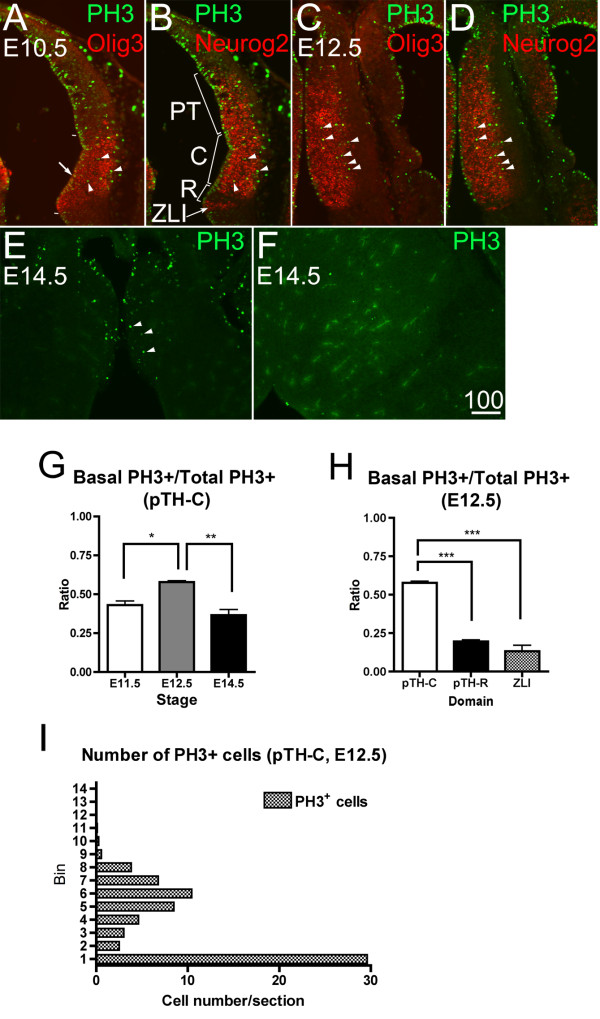
**Numerous cells divide away from the third ventricle in the embryonic mouse thalamus**. **(A-F) **Frontal sections of embryonic day (E)10.5 (A,B), E12.5 (C,D) and E14.5 (E,F) of wild-type mouse forebrain. Midline is to the left. Panels (A,B) and (C,D) each shows the same sections of triple immunohistochemistry for PH3, Olig3 and Neurog2. Panel (E) is more dorsal to panel (F). Numerous PH3-positive cells exist not only at the surface of the third ventricle (A, arrow) but also away from the ventricle (A-D, arrowheads). At E14.5, when thalamic neurogenesis is almost over, a small number of PH3-positive cells are found in dorsal sections (E), but not in more ventral sections (F). Scale bar: 100 μm. PT, pretectum; C, thalamic progenitor domain pTH-C; R, thalamic progenitor domain pTH-R; ZLI, zona limitans intrathalamica. **(G-I) **Cell count of PH3-positive cells in the thalamus. (G) Proportion of PH3-positive cells located more than 40 μm away from ventricular surface (basal PH3^+ ^cells) was calculated for E11.5, E12.5 and E14.5. The ratio of basal PH3^+^/total PH3^+ ^cells was significantly higher at E12.5 than E11.5 or E14.5 (*P < 0.05 and **P < 0.01, respectively; n = 4 for E11.5, E12.5; n = 6 for E14.5). (H) At E12.5, a significantly higher ratio of basal PH3^+^/total PH3^+ ^cells was found in pTH-C compared to pTH-R and ZLI domains (***P < 0.001; n = 4 for pTH-C; n = 6 for pTH-R and ZLI) (I) Distribution of PH3-positive cells in each of the 20-μm-wide medio-lateral bins, showing two distinct populations; one is located closest to the ventricular surface (bin 1) and the other is located more basally, with the peak at bin 6 (mean ± standard error of the mean; 5.0 ± 0.79).

We found numerous cells away from the surface of the third ventricle that express the M-phase marker phosphorylated histone H3 (PH3), which we define as dividing basal progenitor cells (Figure 1A-E, arrowheads). These cells were found as early as at embryonic day (E)10.5 (Figure 1A,B) and persisted until at least E14.5 (Figure 1E). Double/triple immunohistochemistry showed that most of these basal progenitor cells are within the progenitor domain pTH-C, which gives rise to all of the thalamic nuclei that project to the cerebral cortex [19] (Figure 1B, marked as 'C'). Within the pTH-C domain, the ratio of basal PH3-positive cells to total PH3-positive cells was highest at E12.5 and declined at E14.5, when thalamic neurogenesis is largely complete, except in the most dorsal location (Figure 1E-G) [25]. In contrast, fewer PH3-positive cells were found in the progenitor domain pTH-R, which produces neurons that do not project to the cortex [19] (Figure 1B, marked as 'R') and in the ZLI, the border cell population abutting the thalamus and the prethalamus (Figure 1B, marked as 'ZLI'). The ratio of PH3-positive cells in the basal location to the total PH3-positive cells was significantly higher in pTH-C than the other two domains analyzed (pTH-R and ZLI; Figure 1H). Figure 1I shows the average number of PH3-positive cells in each of the 20 μm-wide medial-lateral bins within pTH-C at E12.5. In addition to the high peak at the ventricular (apical) surface (bin 1), there was another peak of PH3-expressing cells away from the third ventricle (bin 6), indicating the presence of a discrete population of thalamic progenitor cells (Figure 1I). These initial analyses demonstrate the presence of basally dividing progenitor cells in the thalamus throughout neurogenesis and that they are particularly enriched in the progenitor domain pTH-C.

### The embryonic mouse thalamus has a defined subventricular zone

We next asked if the basally dividing cells comprise a distinct zone in the mouse thalamus that is not populated by RGs. Such a zone, the SVZ, emerges in mice by E14.5 in the neocortex, and by E13.5 in the ganglionic eminences [26]; however, it has not been evaluated in other brain regions. We used NICD (intracellular domain of Notch) and Pax6 as markers of RGs. NICD is a cleavage product of Notch1 [27], which is co-expressed with Nestin within the neocortical VZ, but not in the SVZ [28]. Notch activity inhibits the formation of IPCs from RGs in the neocortex, indicating that the presence of NICD is a marker for RGs within the VZ. Pax6 is also highly expressed in neocortical RGs in the VZ [29], although low-levels of Pax6 expression are detectable in many IPCs [29] and a recent report identified a new class of Pax6-expressing progenitor cells that divide away from the lateral ventricle in the mouse neocortex [30].

**Figure 2:**
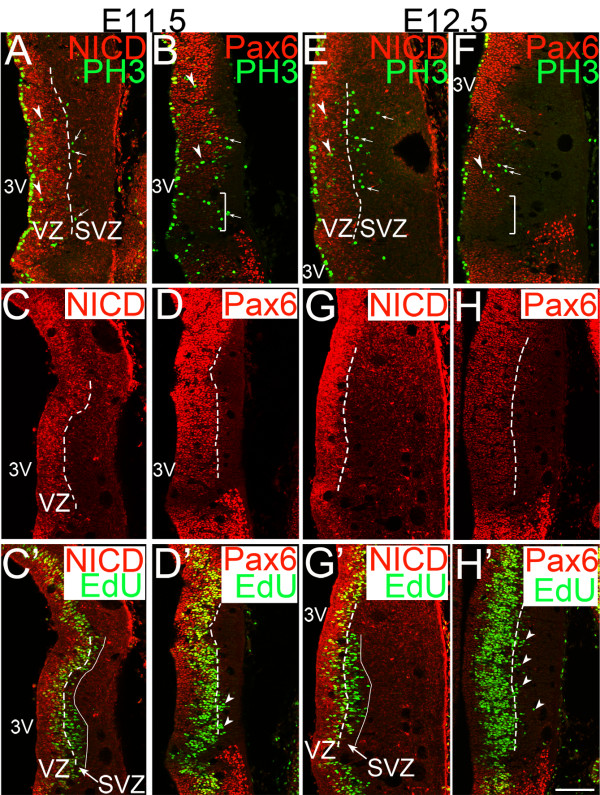
**A subventricular zone exists in the embryonic mouse thalamus**. **(A-H') **Confocal images of frontal sections of E11.5 (A-D,C',D') and E12.5 (E-H,G',H') mouse forebrain showing combined immunostaining of NICD (intracellular domain of Notch1) or Pax6 with 0.5-hour pulse-labeled ethynyl deoxyuridine (EdU). Midline is to the left. Panels (C) and (C'), (D) and (D'), (G) and (G') as well as (H) and (H') are from the same section, respectively. Dashed lines in (A,C,C',E,G,G') show the lateral border of NICD-expressing cells, and dashed lines in (D,D',H,H') show the lateral border of cells with strong Pax6 expression. Solid lines in (C',G') show the lateral border of EdU-expressing cells. The space between the dashed line and the solid line in (C',G') represents the thalamic subventricular zone (SVZ). PH3-positive cells shown by arrowheads (A,B,E,F) indicate dividing cells within the VZ, whereas those shown by arrows (A,B,E,F,D',H') are located outside the VZ within the SVZ. Scale bar: 100 μm. 3V, third ventricle; SVZ, subventricular zone; VZ, ventricular zone.

We found that NICD is expressed in a cluster of cells near the third ventricle at both E11.5 (Figure 2A,C,C', left of dashed line) and E12.5 (Figure 2E,G,G', left of dashed line). Basal PH3-positive cells were located on both sides of the lateral margin of this NICD cluster at E11.5 and E12.5 (Figure 2A,E; arrows indicate the outside population and arrowheads indicate the inside population). The outer population became more evident at E12.5 (Figure 2E). PH3-positive cells were also observed on both sides of the Pax6 domain (Figure 2B,F, arrowheads and arrows). Similar to the neocortex, more laterally located basal progenitor cells expressed low levels of Pax6 (Figure 2B,D,D' at E11.5; 2F,H,H' at E12.5). In addition, Pax6 expression was generally lower in the rostral part of the pTH-C domain at both E11.5 and E12.5 (Figure 2B,F, bracket). Labeling of S-phase cells with a 0.5-hour ethynyl deoxyuridine (EdU) pulse showed that some thalamic progenitor cells reside outside the NICD^+^/Pax6-high zone (Figure 2C',D'G'H'). Based on these results, we propose that, as early as E11.5, a molecularly distinguishable SVZ exists in the pTH-C domain of the thalamus, which we define as the zone where progenitor cells exist outside of the NICD^+^/Pax6-high VZ. Thalamic basal progenitor cells populate both the VZ and SVZ.

### Thalamic and neocortical basal progenitor cells share some molecular properties

**Figure 3:**
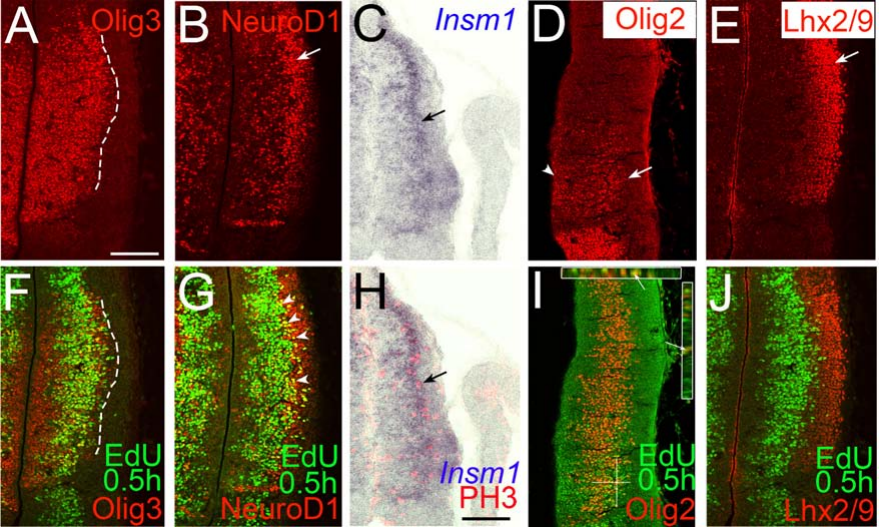
**Expression of various transcription factors in thalamic progenitor cells**. **(A,B,D-G,I,J) **Confocal images of E11.5 frontal sections showing combined immunostaining of Olig3 (A,F), NeuroD1 (B,G), Olig2 (D,I), Lhx2/9 (E,J) with 0.5-hour pulse-labeled EdU. Midline is to the left. (A,F) The dashed line delineates the border of Olig3 expression, which coincides with the lateral border of EdU-expressing cells. (B,G) The arrow in (B) shows the lateral cluster of NeuroD1-expressing cells. The arrowheads in (G) show cells co-expressing NeuroD1 and EdU, which are found within the lateral cluster of NeuroD1-expressing cells. **(C,H) **Combined *in situ *hybridization for Insm1 and PH3 shows overlapping expression of these two markers (arrow). (D,I) The arrowhead and arrow point to the location of Olig2 expression in the VZ and more lateral locations, respectively. Z-projections along the indicated lines are shown in the insets in (I), indicating the co-localization of green and red signals. (E,J) The arrow in (E) shows the lateral position of Lhx2/9-expressing cells that show minimum overlap with 0.5-hour EdU. Scale bar: 100 μm.

**Figure 4:**
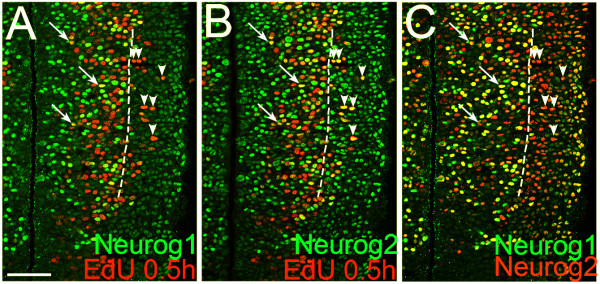
**Comparison of Neurog1 and Neurog2 expression patterns with regards to a 0.5-hour EdU pulse**. **(A-C) **Confocal images of the same E11.5 frontal section showing combined immunostaining of Neurog1 and Neurog2 with a 0.5-hour pulse-labeled EdU on the same section. Midline is to the left. The dashed line delineates the lateral border of Neurog1-expressing cells. Arrows point to cells expressing Neurog1, Neurog2 and EdU within the lateral limit of Neurog1-expressing cells. Arrowheads point to cells that express EdU and Neurog2 but not Neurog1, located outside of the Neurog1-expressing domain. Scale bar: 50 μm.

**Figure 5:**
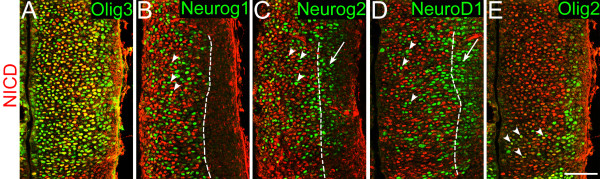
**NICD-expressing radial glial cells also express Olig3 and Olig2, but not Neurog1 or Neurog2**. Confocal images of E11.5 frontal sections showing double immunostaining of NICD and various transcription factors. Midline is to the left. **(A) **Olig3 shows robust overlap with NICD. **(B-D) **The dashed line indicates the lateral border of NICD-expressing cells, which we define as the lateral border of the VZ. Neurog1 (B), Neurog2 (C), and NeuroD1 (D) do not show co-localization with NICD within the VZ (B-D, arrowheads) or within the SVZ (C,D, arrow). **(E) **Arrowheads point to cells co-expressing NICD and Olig2. Scale bar: 50 μm.

We then examined the expression patterns of previously characterized genes that are expressed in thalamic progenitor cells in order to determine the progenitor zone (VZ or SVZ) and progenitor cell types (RGs or basal progenitor cells) in which each gene is expressed (Figures 3, 4 and 5). Thalamic progenitor cells ubiquitously express the bHLH transcription factor Olig3 [19], but the neocortex does not. Double staining with a 0.5-hour EdU pulse showed that the domain of Olig3 expression in the thalamus encompassed the entire medial-lateral extent of thalamic progenitor cells, indicating that Olig3 is expressed in both the VZ and SVZ of the thalamus (Figure 3A,F). In addition, we found that Olig3 heavily overlaps with NICD (Figure 5A), demonstrating that Olig3 is expressed in RGs. Together, these results show that Olig3 is expressed in both the VZ and SVZ and in both RGs and basal progenitor cells in the thalamus.

We next determined if NeuroD1 and *Insm1 *(insulinoma-associated 1), markers for neocortical IPCs, are also expressed in the thalamus. NeuroD1 is a bHLH transcription factor that is expressed in the upper SVZ and lower intermediate zone of the neocortex, presumably being induced following Tbr2 expression [31]. In the pTH-C domain of the thalamus, a densely packed population of NeuroD1-positive cells was found in the middle portion of the diencephalic wall (Figure 3B, arrow). In addition, some NeuroD1-expressing cells were scattered within the VZ. Double immunostaining for NeuroD1 and a 0.5-hour pulse of EdU showed that NeuroD1 is expressed in basally located progenitor cells in S phase of the cell cycle (Figure 3G, arrowheads). These NeuroD1^+^/EdU^+ ^cells seemed to be predominantly located in the SVZ. NeuroD1 was also clearly expressed outside of the NICD-expressing VZ (Figure 5D, arrow) and the scattered NeuroD1-positive cells within the VZ did not express NICD (Figure 5D, arrowheads), indicating the lack of NeuroD1 expression in RGs. Double staining experiments also showed that some basal progenitor cells co-expressed NeuroD1 and PH3 (not shown). These results together demonstrate that NeuroD1 is expressed in thalamic basal progenitor cells at least through S phase to M phase of the cell cycle, but not in NICD-expressing RGs.

Insm1 is a zinc-finger transcription factor expressed broadly in progenitor cells within the embryonic brain and spinal cord located away from the ventricular surface [32]. It is required for the generation of basal progenitor cells in the neocortex [12]. We found that *Insm1 *is strongly expressed in a lateral band of cells within the thalamus. Comparison of *Insm1 *with PH3 on the same section shows that *Insm1 *is indeed expressed in thalamic basal progenitor cells (Figure 3C,H).

Olig2 is a bHLH transcription factor expressed in the pTH-C domain of the thalamus in a rostro-ventral high to caudo-dorsal low gradient at E11.5 and E12.5 [19]. We found that Olig2 is not only expressed in the VZ (Figure 3D, arrowhead) but also in a more lateral region (Figure 3D, arrow). Olig2 expression overlapped with a 0.5-hour EdU pulse (Figure 3I), and extended further laterally (Figure 3I, arrow; Figure 5E). Within the VZ, Olig2 co-localized with NICD (Figure 5E, arrowheads), suggesting that it is expressed in RGs. Thus, similar to Olig3, Olig2 is expressed in both RGs and basal progenitor cells. Olig2 also appeared to be expressed lateral to the SVZ, most likely in the mantle zone.

Finally, Lhx2 and Lhx9 are LIM-homeodomain transcription factors expressed in the thalamus [33,34]. In the neocortex, Lhx2 is expressed in neural progenitor cells and Lhx9 is expressed in the marginal zone [35,36]. We found Lhx2/9-positive cells are largely confined outside the VZ, with only a minimum overlap with a 0.5-hour EdU pulse (Figure 3J), indicating that they are expressed mostly in postmitotic cells.

Interestingly, a well-established IPC marker in the neocortex, Tbr2, a T-box transcription factor [15,29], was undetectable in the thalamus at E11.5 and E12.5 (data not shown).

These results collectively show that although the thalamus has a histologically identifiable SVZ populated by basal progenitor cells and these cells share expression of some genes, such as *Insm1 *and *NeuroD1*, with neocortical IPCs, they are clearly distinct from their putative neocortical counterpart. Thalamic basal progenitor cells do not express Tbr2 and express additional markers such as Olig2 and Olig3 that are not expressed in the neocortex.

### Proneural bHLH proteins Neurog1 and Neurog2 are expressed in overlapping but different progenitor populations in the thalamus

To further characterize the thalamic basal progenitor cells, we examined the expression of two bHLH proteins, Neurog1 and Neurog2, both of which are expressed in neocortical progenitor cells. In the neocortex, expression of Neurog2 is initiated soon after the division of RGs, preceding the induction of Tbr2 [15]. Britz *et al*. [37] reported that at E12.5, 95% of Neurog1-expressing progenitor cells in the cortical VZ also express Neurog2, and at E15.5, both Neurog1 and Neurog2 are expressed in the VZ as well as the SVZ.

We previously showed that Neurog1 and Neurog2 are expressed in the pTH-C thalamic progenitor domain [19]. In this study, we examined the patterns of their expression in more detail. Comparison of Neurog1 expression with EdU (0.5-hour pulse) indicated that Neurog1 expression does not extend as laterally as EdU (Figure 4A, arrowheads), although Neurog1 and EdU overlap at the lateral part of the Neurog1 expression domain (Figure 4A, arrows). Neurog2 expression extended more laterally than Neurog1, where it heavily overlapped with EdU (Figure 4B, arrowheads). Neurog2 also overlapped with EdU more medially, in the region where Neurog1 is expressed (Figure 4B, arrows). A direct comparison of Neurog1 and Neurog2 expression indicated that Neurog2 expression extends more laterally than Neurog1 (Figure 4C, arrowheads). The lateral border of the Neurog1 expression domain matched the lateral border of NICD expression (Figure 5B, dashed line). Thus, in contrast to the neocortex, Neurog1 expression in the thalamus is confined to the VZ. Within the VZ, Neurog1 and Neurog2 showed partially overlapping but distinct expression patterns (Figure 4C). Similar to the neocortex [28], neither of these two transcription factors co-localized with NICD within the VZ (Figure 5B,C, arrowheads). This result is consistent with the hypothesis that neurogenin-expressing VZ cells are basal progenitors translocating laterally towards the SVZ. In contrast, Olig2 and Olig3 were expressed in both the thalamic VZ and SVZ and had extensive overlap with NICD within the VZ (Figure 5A,E).

### Cell cycle properties of basal progenitor cells in the thalamus

**Figure 6:**
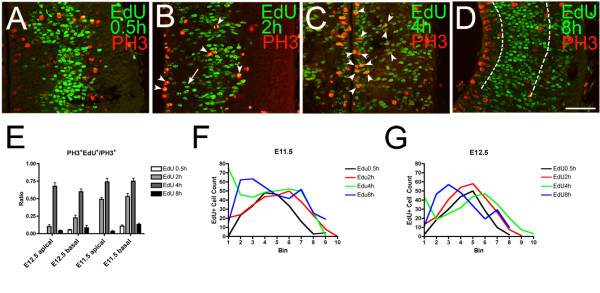
**EdU pulse labeling and PH3 expression**. **(A-D) **Confocal images of E11.5 frontal sections showing double staining of PH3 and EdU pulsed for different durations. Midline is to the left. (A) With a 0.5-hour EdU pulse, PH3 shows no co-localization with EdU in both apically and basally dividing cells. (B) With a 2-hour EdU pulse, some cells show co-localization in both apical and basal locations (arrowheads). Compared with the 0.5-hour pulse, more EdU-labeled cells are found close to the ventricle (arrow). (C) With a 4-hour EdU pulse, many cells express both PH3 and EdU both apically and basally (arrowheads). (D) With an 8-hour EdU pulse, no co-localization of EdU and PH3 is detected. Dashed lines indicate the large, central cluster of EdU-labeled cells. **(E) **Cell counts showing the proportion of PH3^+^/EdU^+ ^cells in total PH3^+ ^cells at E11.5 and E12.5. The greatest proportion of co-localization is found with a 4-hour pulse, suggesting that most cells enter M phase within 4 hours after S phase. Cell counts are shown as mean ± standard error of the mean; one-way ANOVA test F = 27.70, *P *< 0.0001. Post-hoc Tukey test shows significant difference between EdU 0.5 hours versus 2 hours (*P *< 0.01), EdU 0.5 hours versus 4 hours (*P *< 0.001), EdU 2 hours versus 4 hours (*P *< 0.05), EdU 2 hours versus 8 hours (*P *< 0.01), and EdU 4 hours versus 8 hours (*P *< 0.001) at E11.5 and a significant difference between EdU 0.5 hours versus 4 hours (*P *< 0.01), EdU 2 hours versus 4 hours (*P *< 0.01), and EdU 4 hours versus 8 hours (*P *< 0.01) at E12.5. No significance is detected for ratios between apically and basally dividing cells at the same pulse time. Scale bar: 50 μm. **(F,G) **Distribution of EdU-positive cells for each pulse time at E11.5 (F) and E12.5 (G). Each bin is 20-μm wide and bin 1 is at the surface of the third ventricle. Three sections for each pulse time at each stage were averaged.

We next examined the cell cycle properties of thalamic basal progenitor cells. First, we pulsed the progenitor cells with an S-phase marker, EdU, and analyzed the distribution of PH3-positive cells at various times after EdU injection. We detected EdU and PH3 on the same section of E11.5 and E12.5 embryos to estimate the time it takes progenitor cells to enter M phase (Figure 6). In E11.5 embryos that had been pulsed with EdU 0.5 hours prior to sacrifice, we detected a large, single cluster of EdU-positive cells that encompassed a broad medial-lateral region of the thalamic progenitor domain, suggesting the close proximity of RGs and basal progenitor cells during S phase (Figure 6A; black curve in Figure 6F,G). As expected, very few mitotic cells expressing PH3 are labeled by EdU.

At 2 hours after EdU injection, we detected some EdU-positive cells at the ventricular surface and the region closer to the ventricle (Figure 6B, arrow; red curve in Figure 6F,G). Many PH3-positive cells both at the ventricular surface and in the basal location were also EdU-positive (Figure 6B, arrowheads). This indicates that, particularly at E11.5, cells start to enter M phase about 2 hours after S phase.

At 4 hours, as many as 60 to 75% of PH3-expressing cells were positive for EdU at both the apical and basal locations (Figure 6C, arrowheads; Figure 6E). In addition, we found two dense clusters of EdU-positive cells that were now separated from each other. One was located close to the ventricle. The other population was located more laterally (green curve in Figure 6F,G). This separation implies a distinct migratory behavior of thalamic basal progenitor cells, which stay in the basal location from S phase to M phase. Conversely, RGs translocate their nuclei medially from S phase to M phase by interkinetic nuclear migration.

At 8 hours, we again detected only a small overlap between EdU and PH3, indicating that a majority of progenitor cells labeled 8 hours before have already divided. A broad cluster of EdU-positive cells was found in the middle of the diencephalic wall (Figure 6D, between the dashed lines), and additional EdU-positive cells were found far laterally, which are likely to be postmitotic cells (blue curve in Figure 6F,G).

In summary, the EdU pulse experiment distinguishes RGs and basal progenitor cells because of their distinct patterns of migration during their cell cycle.

### Expression of basal progenitor markers at different stages of the cell cycle

By taking advantage of the EdU pulse labeling, we next examined the expression of NeuroD1, Lhx2/9, Neurog1 and Neurog2 in more detail with regard to the cell cycle status of basal progenitor cells.

**Figure 7:**
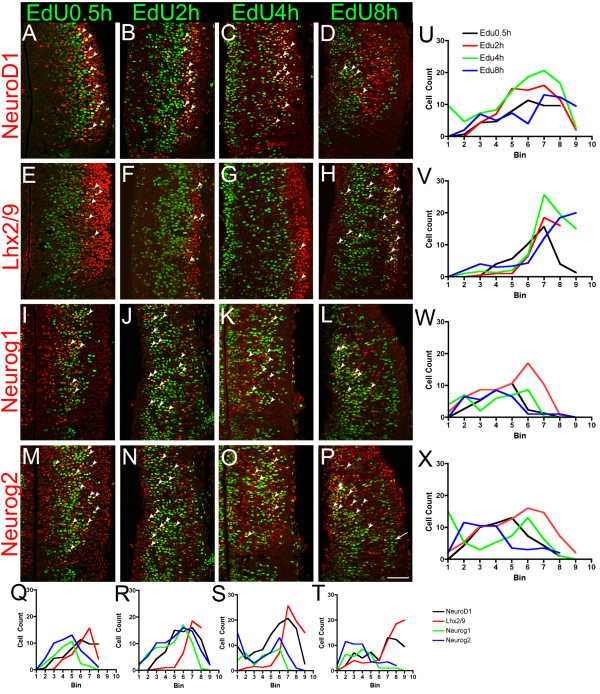
**Co-expression of various transcription factors with EdU at different pulse durations**. Confocal images of E11.5 frontal sections showing double staining of various transcription factors and EdU pulsed for different durations of time. Midline is to the left. **(A-D) **NeuroD1 is co-localized with EdU in basal locations at 0.5 hours, 2 hours, 4 hours and 8 hours after EdU labeling (arrowheads). **(E-H) **Lhx2/9 shows co-localization with EdU mostly at lateral locations with 0.5-hour, 2-hour and 4-hour pulses (arrowheads), but with an 8-hour pulse, scattered EdU-positive cells in the VZ as well as in far lateral locations show co-localization. **(I-L) **With a 0.5-hour pulse, Neurog1 is co-expressed with EdU near the lateral margin of the VZ (arrowheads). With 2-hour and 4-hour pulses, more double-positive cells are located medially, but not at the ventricular surface (arrowheads). **(M-P) **Neurog2 shows a similar pattern to that of Neurog1, but cells expressing Neurog2 and EdU are found more laterally than Neurog1+/EdU+ cells (arrowheads). Neither of the neurogenins shows robust co-localization with EdU with an 8-hour pulse in lateral locations (P, arrow). Scale bar: 50 μm. **(Q-T) **Cell counts for each EdU pulse time are shown for each marker. Three sections for each sample were counted and cell numbers are averaged. Each bin is 20-μm wide and bin 1 is at the surface of the third ventricle. **(U-X) **Cell counts for each marker at different EdU pulse times. Three sections for each sample were counted and cell numbers are averaged. Each bin is 20-μm wide and bin 1 is at the surface of the third ventricle.

**Figure 8:**
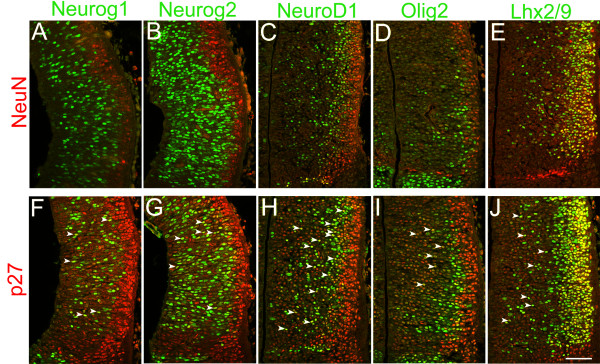
**Co-expression of various transcription factors with postmitotic neuronal markers**. Confocal images of E11.5 frontal sections showing double staining of various transcription factors and NeuN (A-E) or the cyclin-dependent kinase inhibitor p27 (F-J). Midline is to the left. **(A-D) **Neurog1, Neurog2, NeuroD1 and Olig2 show almost no overlap with the postmitotic neuronal maker NeuN. NeuN-expressing cells are restricted to the lateral part of the section. **(E) **Lhx2/9 and NeuN are co-expressed. **(F-I) **Neurog1, Neurog2, NeuroD1 and Olig2 show some co-expression with the cyclin-dependent kinase inhibitor p27 in the VZ and SVZ (arrowheads). **(J) **Abundant co-localization of Lhx2/9 and p27 in both the VZ/SVZ (arrowheads) and the mantle zone. Scale bar: 50 μm.

As already shown in Figure 3, NeuroD1 was co-localized with 0.5-hour EdU only in the basal location (Figure 7A; Figure 7U, black line). Co-localization of NeuroD1 with EdU continues in the basal location at 2 hours (Figure 7B, arrowheads; Figure 7U, black curve), 4 hours (Figure 7C, arrowheads; Figure 7U, green curve) and 8 hours (Figure 7D, arrowheads; Figure 7U, blue curve), indicating that basal progenitor cells express NeuroD1 throughout their cell cycle after the newly generated cells reach the basal location. NeuroD1 also partially co-localized with p27 (Figure 8H), a cyclin-dependent kinase inhibitor expressed in differentiating neural progenitor cells as well as postmitotic neurons [38,39], but it did not co-localize with NeuN (Figure 8C), a marker for a subset of postmitotic neurons, suggesting that NeuroD1 expression is transient.

Lhx2/9 was expressed in the lateral part of the thalamus, and showed only a minor overlap with EdU at each of the pulse times (Figure 7E-H,7V). The overlap with neuronal markers NeuN and p27 was robust (Figure 8E,J), indicating that Lhx2/9 expression persists in postmitotic neurons, consistent with a previous study showing widespread expression of Lhx2 and Lhx9 in postmitotic thalamic nuclei [33].

As shown in Figure 4, Neurog1 is co-localized with 0.5-hour EdU in the VZ (Figure 7I, arrowheads; Figure 7W, black curve) but not in the SVZ. In contrast, Neurog2 co-localization with 0.5-hour EdU was found in both the VZ and SVZ (Figure 7M, arrowheads; Figure 7X, black curve). Cells co-expressing EdU and Neurog1 and cells co-expressing EdU and Neurog2 were found more medially with a 2-hour pulse (Figure 7J,N, arrowheads; Figure 7W,X, red curve). At 4 hours, when most PH3-positive cells are also EdU-positive, more cells co-expressing neurogenins and EdU were found near the third ventricle, in addition to the lateral cluster (Figure 7K,O, arrowheads; Figure 7W,X, green curve). With an 8-hour pulse, we detected a single cluster of double-labeled cells, while the lateral cluster of EdU-positive cells, which are likely to be postmitotic neurons, did not express neurogenins (Figure 7L,P, arrowheads; Figure W,X, blue curve). These results indicate that Neurog1 and Neurog2 are both induced as newly formed basal progenitor cells migrate to basal positions, within either the VZ (for both Neurog1 and Neuorg2) or the SVZ (for Neurog2), and their expression is maintained as the basal progenitor cells undergo cell cycles and divide again. Double staining with NeuN (Figure 8A,B) and p27 (Figure 8F,G) showed that neurogenins overlap with p27 but not with NeuN. Thus, the expression of neurogenins is transient.

### Neurogenins are required for the formation and/or maintenance of basal progenitor cells in the thalamus

In the neocortex, Neurog1 and Neurog2 together play a role in neuronal differentiation and, at the same time, in the specification of the dorsal telencephalic fate of neural progenitor cells [40]. Microarray analysis shows that the expression levels of *Tbr2 *and *NeuroD1 *in the neocortex are decreased in *Neurog1/2 *double knockout mice [40]. Although histological analysis of cortical IPCs with immunohistochemistry for Tbr2 and NeuroD1 has not been reported in these mutant mice, both PH3-positive mitotic cells and bromodeoxyuridine-labeled S-phase progenitor cells are increased in the SVZ and decreased in the VZ in *Neurog2 *single as well as *Neurog1/2 *double knockout mice [37], suggesting that these transcription factors are likely to play an important role in IPC specification and/or differentiation.

**Figure 9:**
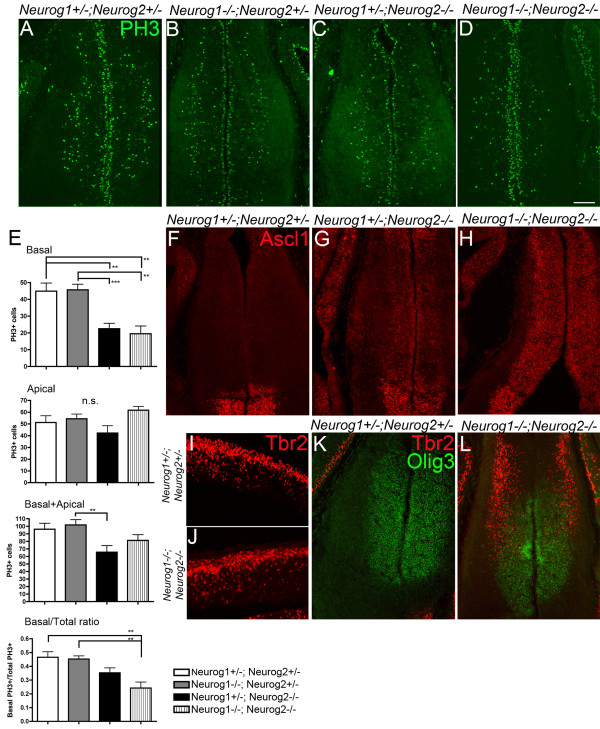
**Neurogenins are required for the normal number of basally dividing progenitor cells in the thalamus**. **(A-D) **E12.5 frontal sections showing the expression of the mitosis marker PH3 in (A) control compound heterozygote (*Neurog1^-/-^;Neurog2^+/-^*), (B) *Neurog1 *single knockout (*Neurog1^+/-^;Neurog2^-/-^*), (C) *Neurog2 *single knockout (*Neurog1^+/-^;Neurog2^-/-^*) and (D) *Neurog1/2 *double knockout (*Neurog1^-/-^;Neurog2^-/-^*) embryos. **(E) **Cell count of PH3-positive cells in the pTH-C domain of the thalamus of neurogenin knockout mice (n = 9 for *Neurog1^+/-^;Neurog2^+/-^*, n = 13 for *Neurog1^-/-^;Neurog2^+/-^*, n = 13 for *Neurog1^+/-^;Neurog2^-/-^*, n = 4 for *Neurog1^-/-^;Neurog2^-/-^*). One sample is one section. Numbers of basal, apical and basal plus apical PH3-positive cells as well as the ratio of basal PH3^+ ^cells/total PH3^+ ^cells were compared between the genotypes. One-way ANOVA for *Neurog1^+/-^;Neurog2^+/-^*, *Neurog1^-/-^;Neurog2^+/-^*and *Neurog1^+/-^;Neurog2^-/- ^*embryos; F = 11.96 for basal PH3, F = 1.744 for apical PH3, F = 4.517 for basal + apical PH3, F = 5.881 for basal/total ratio. N.s., not significant; **P < 0.01. **(F-H) **Expression of the pTH-R/prethalamic marker Ascl1 in neurogenin knockout mice at E12.5. Note induced expression of Ascl1 in the pTH-C domain in (G,H). **(I-L) **Expression of transcription factor Tbr2 in *Neurog1/2 *double knockout embryos at E12.5. Panels (I,J) show reduced but still persistent expression of Tbr2 in the neocortex of *Neurog1/2 *double knockout mice. (K,L) Induction of Tbr2 in the thalamic mantle zone of *Neurog1/2 *double knockout mice. Olig3 is used as a reference for the thalamic VZ and SVZ. Scale bar: 100 μm for (A-D,F-L).

In order to determine if neurogenins play a role in the formation or maintenance of basal progenitor cells in the thalamus, we analyzed *Neurog1/2 *double knockout mice and *Neurog1 *and *Neurog2 *single knockout mice in comparison with double heterozygous controls. We found that double knockout mice (*Neurog1^-/-^*; *Neurog2^-/-^*) have fewer PH3-positive, dividing basal progenitor cells in the pTH-C domain at E12.5 (Figure 9D,E). Both the absolute number and the ratio against the total PH3-positive cell number were significantly reduced from the controls. In contrast, the number of apical PH3-positive cells or the total PH3-positive cells did not show a significant difference. The *Neurog2 *single (*Neurog1^+/-^*; *Neurog2^-/-^*) mutant showed reduction in absolute number of basal PH3-positive cells but not in the ratio against the total PH3-positive cells (Figure 9C,E). The *Neurog1 *single mutant did not show any significant difference from the control (Figure 9B,E). These results indicate that neurogenins are required for the normal number of basally dividing progenitor cells in the thalamus, and that the role of Neurog2 is only partially compensated by Neurog1.

As already shown previously [41], another bHLH transcription factor, Ascl1 (also known as Mash1) is induced in the neocortex of *Neurog2 *single and *Neurog1/2 *double mutant mice. Ascl1 is normally expressed at a high level in the ventral telencephalon, suggesting a role for neurogenins in specifying dorsal telencephalic fate and suppressing ventral telencephalic fate. It has also been shown that neurogenins are required to suppress Ascl1 expression in the thalamus [41,42]. Consistent with these previous findings, we found robust Ascl1 induction in the thalamus of *Neurog1/2 *double mutant mice (Figure 9H), whereas *Neurog2 *single mutants (*Neurog1^+/-^*; *Neurog2^-/-^*) showed much less severe induction of Ascl1 (Figure 9G). Ascl1 was not induced in *Neurog1 *single mutants (*Neurog1^-/-^*; *Neurog2^+/-^*; data not shown). These results demonstrate that neurogenins, of which Neurog2 is the prominent one, suppresses Ascl1 expression. Reduction of the basal progenitor cell number in the thalamus of neurogenin mutant mice indicates that Ascl1 does not compensate for the function of neurogenins in this cell type. Interestingly, Tbr2, a cortical IPC marker, was normally not expressed in the thalamus but was ectopically induced in the mantle zone of the thalamus of the *Neurog1/2 *double mutant (Figure 9K,L). Considering the fact that SVZ mitosis was increased in the neocortex [37] but decreased in the thalamus (Figure 9) of *Neurog1/2 *double knockout mice, we conclude that the roles of neurogenins in basal progenitor cells in the thalamus are likely different from those in the neocortex.

**Figure 10:**
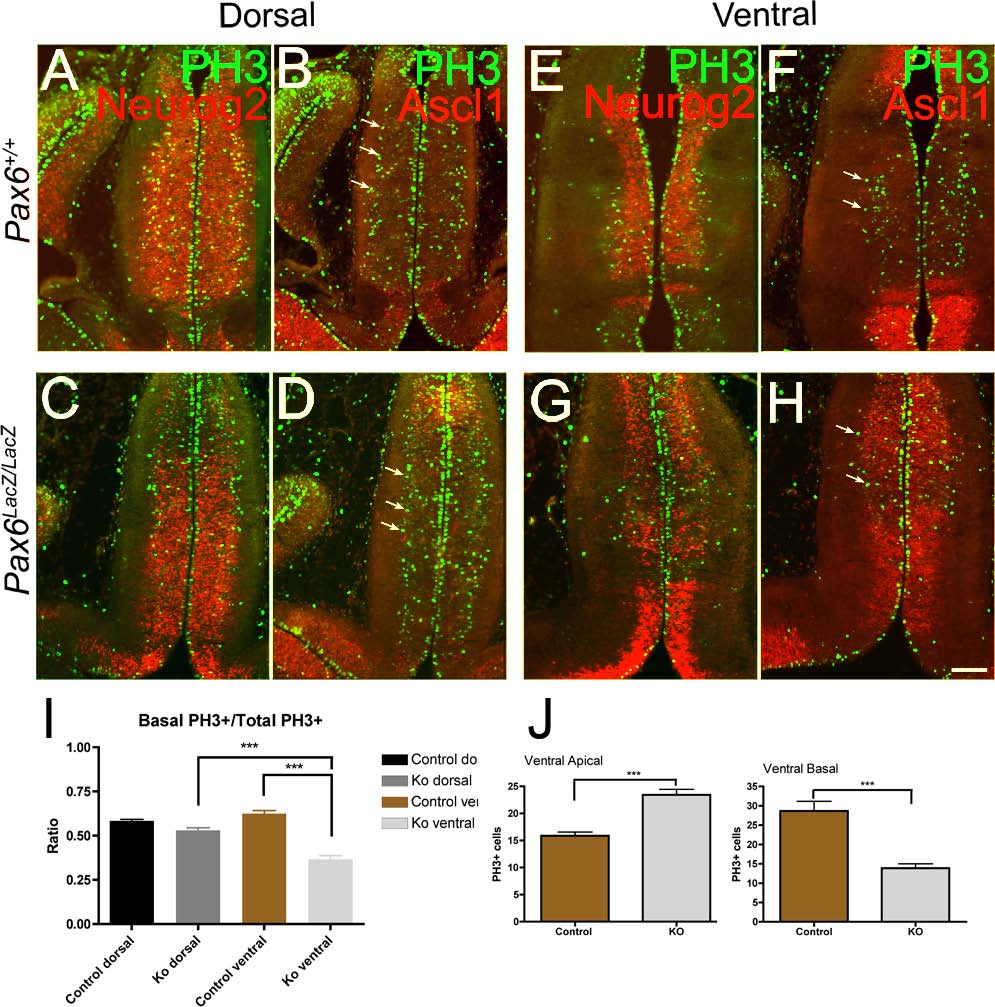
**Pax6 mutant mice show regionally specific mis-expression of Ascl1 and reduction of Neurog2 expression in the thalamus, which is accompanied by reduced basally dividing cells**. E12.5 frontal sections showing double immunostaining of Neurog2 (A,C,E,G) or Ascl1 (B,D,F,H) with PH3 in *Pax6 *mutant embryos (C,D,G,H) and their littermate control (A,B,E,F). **(A-D) **Sections at the dorsal level show a largely normal level of Neurog2 expression in the pTH-C domain of the thalamus (A,C) and the absence of Ascl1 expression (B,D). Basal PH3-positive cells also appear normal (B,D, arrows). **(E-H) **In more ventral sections, however, Neurog2 expression is reduced (E,G) and Ascl1 is ectopically expressed (F,H). In the region where Ascl1 is induced, basal PH3-positive cells appear to be reduced (F,H, arrows). **(I,J) **Cell count of PH3-positive cells in the pTH-C domain of the thalamus of *Pax6 *knockout mice (n = 14 for each dorsal section, n = 14 for ventral sections). One sample is one section. (I) The ratio of basal PH3+ cells/total PH3+ cells was compared between control dorsal, *Pax6 *knockout dorsal, control ventral, and *Pax6 *knockout ventral sections. One-way ANOVA was done to compare; F = 56.25. (J) The numbers of apical and basal PH3-positive cells on ventral sections were compared between the *Pax6 *knockout and the control wild-type embryos. Student's *t*-test was done. ***P < 0.001. Error bars are standard error of he mean (SEM). Scale bar: 100 μm for (A-H).

The paired-/homeo-domain transcription factor Pax6 is known to play a critical role in thalamic development [43]. As already shown in Figure 2, high-level expression of Pax6 was detected in the thalamic VZ, although the expression decreased in the rostro-ventral part of the pTH-C domain at E11.5 and later. In *Pax6 *mutant mice, we detected reduction of Neurog2 expression (Figure 10E,G) and ectopic induction of Ascl1 (Figure 10F,H) in the ventral part of the pTH-C domain, but not in the dorsal part (Figure 10A-D). The ratio of basal PH3-positive cells was specifically reduced in ventral sections, where a large number of Ascl1-expressing cells were intermingled with Neurog2-expressing cells (Figure 10G-I). The decrease in the number of basal PH3-positive cells was accompanied by an increase in the number of apical PH3-positive cells (Figure 10J), indicating the role of Pax6 in generating basal progenitor cells from apical progenitor cells. The total number of basal plus apical PH3-positive cells did not change between wild-type and mutant embryos, at both dorsal and ventral levels (data not shown).

## Discussion

In this study, we showed that, throughout thalamic neurogenesis, a high proportion of progenitor cells divide away from the third ventricle and some of these basal cell divisions occur outside of the VZ. We found that basal progenitor cells are most abundant in the thalamic progenitor domain that expresses the bHLH transcription factors Neurog1 and Neurog2, which are also expressed in the neocortex where basal progenitor cells abound. The thalamus and the neocortex share some of the molecular markers expressed in these cell populations, including Neurog1, Neurog2, NeuroD1 and *Insm1*, but each also expresses a unique set of genes. For example, Tbr2 is expressed only in the neocortex and Olig2 and Olig3 are expressed only in the thalamus. We then characterized various transcription factors that are differentially expressed at different cell cycle stages of thalamic progenitor cells. We further showed that two bHLH transcription factors, Neurog1 and Neuorg2, as well as the paired-/homeo-domain transcription factor Pax6, are required for the normal number of thalamic basal progenitor cells.

### Medial-lateral organization of the thalamic progenitor domain

**Figure 11:**
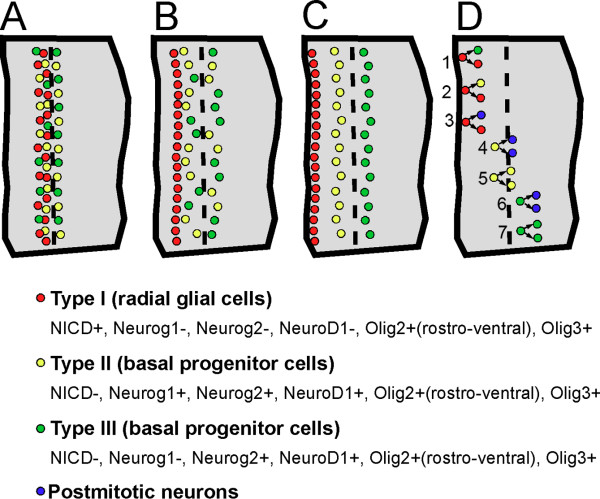
**Summary of distinct progenitor cell populations in the thalamus - a working hypothesis**. Based on our observations, we propose a working hypothesis in which three types (types I, II, and III) of progenitor cells reside in the embryonic mouse thalamus. A to C denote the position of these progenitor cells at different phases of the cell cycle. Midline is to the left. The dashed line indicates the VZ/SVZ border. **(A) **Cells in S phase, most typically detected by a 0.5-hour EdU pulse. All the progenitor cell types comprise a single, wide column in the middle of the thalamus. **(B) **Cells in G2 phase, most typically detected by a 2-hour EdU pulse. RGs are moving towards the ventricle by interkinetic nuclear migration. **(C) **Cells in M phase. RGs divide at the ventricular surface, whereas type II and III cells (both are basal progenitor cells) divide away from the ventricle. Type II cells divide in the VZ and type III cells divide in the SVZ. **(D) **Hypothetical mode of cell division based on studies on neocortical IPCs. RGs divide asymmetrically to produce a RG and either a basal progenitor cell (1,2) or a neuron (3). Type II cells divide basally in the VZ and generate two neurons (4) or two type II cells (5). Type III cells divide basally in the SVZ and generate two neurons (6) or two type III cells (7). The actual lineage relationship between the three progenitor cell types in the thalamus is a topic of future investigation.

Our current study has identified a domain of progenitor cells outside the thalamic VZ. We term this external zone of progenitor cells the thalamic SVZ. The term thalamic SVZ has been used before (for example, [44]), but no reports have shown the presence of progenitor cells outside of the VZ that are either dividing or in S phase of the cell cycle. In this paper, we used the presence of cleaved Notch1 (NICD) as the landmark of the VZ [28]. In the neocortex, Notch signaling plays a critical role in maintaining RG fate and inhibiting the expression of Neurog2 and Tbr2, and thus the formation of IPCs [14,15,45]. Within the pTH-C domain of the thalamus, progenitor cells outside the VZ start to be detectable at E11.5 and become more prominent at E12.5, although basal cell division occurs not only within the SVZ, but also in the VZ as early as E10.5, when thalamic neurogenesis has just started [25]. Based on these results, we propose a classification of thalamic progenitor cells into three types based on where they divide. The first population (type I) is the RGs that divide at the apical surface of the third ventricle. If we define them as producing NICD (although the signal is weaker near the ventricular surface), they also express Olig3 and Olig2. The second (type II) and third (type III) populations divide away from the ventricular surface; type II cells divide within the VZ and type III cells divide in the SVZ. Based on our gene expression analysis, Neurog1 differentiates type II and type III cells because it is expressed by NICD-negative cells in the VZ, but not in the SVZ. Among the markers expressed in both types of progenitor cells, Neurog2 is expressed evenly between the VZ and SVZ, while NeuroD1-expressing cells are distributed much more densely in the SVZ than the VZ. Two other bHLH factors, Olig2 and Olig3, are expressed in all three progenitor types (summarized in Figure 11). Interestingly, a recent study analyzed gene expression profiles of single progenitor cells in E14 mouse cortex and classified the progenitor cells into three clusters that likely correspond to RGs, VZ basal progenitors and SVZ basal progenitors [45]. Since it is unclear whether the two basal progenitor cell clusters in the cortex represent the difference in their state of cell cycle phase (for example, G1/S phases for VZ basal progenitor cells and G2/M phases for SVZ progenitor cells) or the location of mitosis, it remains to be determined how our two progenitor populations (types II and III) differ in overall gene expression profiles and how they are related with regard to cell lineage.

### What is the significance of the large number of basal progenitor cells in the thalamus?

Our current study shows that the thalamus is one of the brain regions where basal progenitor division is most prominent. In comparison with a similar quantification study, the ratio of basal PH3-positive cells in E12.5 thalamus is comparable to the peak ratio of basal divisions in neonatal cortex [46]. As in the neocortex, basal progenitor divisions occur in both the VZ and SVZ. Although basal division of neural progenitor cells has been described in many regions in the central nervous system, including the spinal cord and the hindbrain [5-9], the existence of the embryonic SVZ, which is populated exclusively by basal progenitor cells and not by RGs, has been described only for the neocortex and the ventral telencephalon, where basal progenitor cells are dominant and a large number of neurons are generated. We propose that the thalamus belongs to this group of brain regions. Considering the extensive interconnections between the mammalian thalamus and the neocortex, it is intriguing to speculate that these two brain regions have evolved together to produce a balance in the large numbers of neurons needed to connect these regions.

Recent lineage tracing studies show that both Neurog1- and Neurog2-expressing progenitor cells produce neurons of all thalamic nuclei that project to the neocortex [19,47]. Because Neurog1 is expressed in basal progenitor cells in the VZ but not in the SVZ, whereas Neurog2 is expressed in both populations, it will be interesting to determine if the basal progenitor cells in the VZ and those in the SVZ generate different sets of neurons in each nucleus. The potentially distinct postmitotic cell fates of Neurog1- and Neurog2-expressing progenitor cells might result in specific thalamic phenotypes in *Neurog2 *single knockout mice. Seibt *et al*. [48] showed normal expression of *Lhx2 *and *Gbx2*, both of which are widely expressed in postmitotic thalamic neurons at E12.5, in *Neurog2 *single knockout mice. We have also obtained similar results in *Neurog1^+/-^*; *Neurog2^-/- ^*mice (data not shown). Thus, analysis of later embryonic stages with nuclei-specific markers would be necessary to reveal the specific roles of Neurog2 in thalamic neurogenesis.

Although our study does not provide information on how the thalamic basal progenitor cells migrate, divide and produce progeny in real time, it is possible they have similar properties to neocortical IPCs, which divide symmetrically to self renew or produce two neurons, and that thalamic basal progenitor cells contribute to the diversity and the large neuronal number of thalamic nuclei. Our analysis of gene expression combined with EdU pulsing support this hypothesis (summarized in Figure 11). It is also possible that some thalamic basal progenitor cells later generate oligodendrocytes and/or astrocytes. Future lineage tracing studies using live imaging and genetic fate mapping will be able to test these possibilities.

### Roles of neurogenins and Pax6 in the generation/maintenance of thalamic basal progenitor cells

We observed a decreased number of basal PH3-positive cells in the thalamus of *Neurog2 *single and *Neurog1/2 *double knockout embryos. In the neocortex, the level of *Tbr2 *mRNA is decreased in neurogenin mutant mice [37], and *in vivo *over-expression of Neurog2 increased Tbr2-expressing cells in the cortex 24 hours after electroporation [15]. However, basal PH3-positive cells were increased at the expense of apical mitosis in the neocortex of *Neurog2 *single and *Neurog1/2 *double knockouts [37]. In contrast, we saw a decrease in basal progenitor cells and an unchanged number of apical progenitor cells in the thalamus of these knockout mice. Our data suggest that Neurog2, once induced in one of the daughter cells after the radial glial division in the neocortex and perhaps in the thalamus, plays a cell-autonomous role in specifying basal progenitor fate [15]. Differences in the genes regulated by neurogenins in the thalamus and neocortex and the functions of these genes may account for the different phenotypes in the SVZ of neurogenin knockout mice.

There are many other regions in the central nervous system where neurogenins are expressed, but, among them, only the neocortex and the thalamus appear to have prominent populations of basally dividing cells. Conversely, the ventral telencephalon expresses Ascl1 and not neurogenins, but still contains a large number of basally dividing progenitor cells. Therefore, expression of neurogenins alone is not sufficient or absolutely necessary for a large population of basal progenitor cells. Further study is needed to determine what other molecules play a shared role in the neocortex and thalamus.

Our study also showed that *Pax6 *mutant mice have reduced numbers of basal progenitor cells in the ventral part of the thalamus where there was a severe reduction of neurogenin expression and massive induction of Ascl1. Unlike in the neurogenin mutants, however, we saw increased apically dividing cells in this region of the thalamus. The similarities and differences between the neurogenin and *Pax6 *mutants indicate that although Pax6 regulates the formation of basal progenitor cells in the thalamus by regulating the normal expression of neurogenins, it may also have distinct roles in RGs, which control the balance between the apical and basal progenitor cells.

## Conclusions

Our study provides evidence for the presence of a prominent population of basal progenitor cells in the embryonic mouse thalamus, part of which forms the SVZ. Combined analysis of transcription factor expression and cell cycle status revealed that these basal progenitor cells may be divided into two populations: one that divides in the VZ and another that divides in the SVZ (summarized in Figure 11 as a working hypothesis). We also found that neurogenins and Pax6 are required for the formation and/or maintenance of basal progenitor cells in the thalamus. Our study implicates the importance of this special progenitor cell population in enhancing the generation of neurons within the thalamus and may also be critical for generating neuronal diversity in this complex brain region.

## Materials and methods

### Animals

Care of and experimentation on mice were done in accordance with the Institutional Animal Care and Use Committee of the University of Minnesota. Noon of the day on which the vaginal plug was found was counted as E0.5. Stages of early embryos were confirmed by morphology [49]. Timed-pregnant CD1/ICR mice (Charles River) were used for gene expression analysis of wild-type mice. *Pax6 *mutant mice [50] were obtained from G Lanuzo and M Goudling at the Salk Institute and were kept in CD1 background. *Neurog1 *[51] and *Neurog2 *[48] mutants were established in F Guillemot's lab (National Institute for Medical Research, London), produced by J Johnson's lab, and were kept in CD1 background.

### Axial and anatomical nomenclature

Axial and anatomical nomenclatures are described in [19]. The two progenitor domains of the thalamus, pTH-C and pTH-R, as well as the ZLI were identified by the expression of marker genes *Olig3*, *Ascl1 *and *Neurog2 *[19].

### Immunohistochemistry

Immunohistochemistry was performed as described [19,30]. Additional antibodies used were: anti-NICD (rabbit, 1:100; Cell Signaling, Danvers, MA, USA), anti-NeuroD1 (goat, 1:100; Santa Cruz, Santa Cruz, CA, USA), anti-PH3 (mouse and rabbit, 1:100; Millipore, Temecula, CA, USA) and anti-Lhx2 (goat, 1:100; Santa Cruz, sc-19344). Antibody sc-19344 appears to detect a broad postmitotic region in the thalamus at E14.5 (data not shown), consistent with the possibility that it recognizes both Lhx2 and Lhx9 [33]. For NICD detection, we extended the boiling time to 10 minutes to enhance the antigen retrieval, and also used a Tyramide Signal Amplification System (Perkin Elmer, Waltham, MA, USA). Detailed protocols for the entire procedures are available upon request.

### *In situ *hybridization

In situ hybridization was performed as described [19]. *Insm1 *cDNA was obtained from J Garcia-Anoveros (Northwestern University).

### Cell cycle analysis

EdU was dissolved at 0.5 mg/ml in PBS, and injected intraperitoneally into pregnant female mice at 10 μg/g body weight. Embryos were dissected after varying amounts of time (0.5 hours, 2 hours, 4 hours or 8 hours). EdU was detected with a protocol based on that reported in [52]. For simultaneous detection of EdU and various other antigens, cryosectioned brains on slides were first treated with primary and secondary antibodies. Slides were then washed once with 1× PBS and permeabilized with 0.5% Triton, then rinsed twice with 1× PBS. EdU labeling was detected with the Click-iT EdU Imaging Kit (Invitrogen, Carlsbad, CA, USA); detection solution was applied directly to slides and incubated for 15 minutes. Slides were then rinsed and coverslipped according to our previous immunostaining protocol [19].

### Image analysis

Images were collected with a Nikon E800 microscope or Olympus FluoView 1000 confocal microscope and assembled by Image J (NIMH) and Photoshop CS3 or CS5 (Adobe).

### Cell counting of PH3- and EdU-expressing cells

For single counts of PH3-expressing cells, images of 20-μm-thick frontal sections were taken with a Nikon E800 fluorescent microscope. The embryonic mouse thalamus was delineated using specific markers characteristic of the pTH-C and pTH-R domains and the ZLI [19]. For each section, the thalamus was divided into 20-μm bins from the ventricular surface to the lateral surface. Counts were taken for PH3-positive cells per bin. The cell counts from all sections were summed for each half brain. The proportion of PH3-positive cells away from the ventricular surface (>40 μm or >2 bins) was calculated from the total number of PH3-positive cells in the thalamus. The average proportion of PH3-positive cells was calculated with four thalami per embryonic stage.

For EdU and PH3 co-localization, images were acquired by an Olympus FluoView 1000 confocal microscope. Each section was divided into bins similar to those described above, and the proportion of PH3-positive cells that co-localized with EdU was calculated from the total PH3-positive cell count. Five to six 3-μm-thick optical slices were obtained for each field of view, and two of them were taken for cell counts.

For EdU-positive cell count and co-localization of EdU and basal progenitor markers, images were acquired and analyzed as described for EdU and PH3 co-localization. However, only a portion of the thalamus (the first 200 μm from the pTH-C/pTH-R border) was analyzed.

For PH3-positive cell count in *Pax6 *mutants, two or three adjacent sections of a 300-μm-long column of the pTH-C domain were analyzed separately for dorsal and ventral levels. The ratio of basal PH3-positive cells was calculated for each of the 14 sections counted for each genotype. In addition, the absolute numbers of PH3-positive cells per section were also counted and compared.

Cell count data were analyzed and graphed using Prism 4 Software (GraphPad). A one-way ANOVA test was used to determine statistical significance, where *P *< 0.05 indicated significance. A post-test - the Tukey multiple comparison's test - was used to determine significance among groups. Double asterisks in indicate *P *< 0.01, triple asterisks indicate *P *< 0.001.

## Abbreviations

bHLH: basic helix-loop-helix; E: embryonic day; EdU: ethynyl deoxyuridine; IPC: intermediate progenitor cell; NICD: intracellular domain of Notch; PBS: phosphate-buffered saline; PH3: phosphorylated histone H3; RG: radial glial cell; SVZ: subventricular zone; VZ: ventricular zone; ZLI: zona limitans intrathalamica.

## Competing interests

The authors declare that they have no competing interests.

## Authors' contributions

LW carried out experiments on wild-type embryos and drafted the manuscript. KKB carried out experiments on neurogenin and *Pax6 *mutants and drafted the manuscript. LD generated and genotyped neurogenin mutant embryos. YN conceived the study, and participated in its design and coordination and wrote the manuscript. All authors read and approved the final manuscript.
